# The *Plasmodium falciparum* Rh5 invasion protein complex reveals an excess of rare variant mutations

**DOI:** 10.1186/s12936-021-03815-x

**Published:** 2021-06-23

**Authors:** Leonard Ndwiga, Victor Osoti, Kevin Omondi Ochwedo, Kevin Wamae, Philip Bejon, Julian C. Rayner, George Githinji, Lynette Isabella Ochola-Oyier

**Affiliations:** 1 KEMRI-Wellcome Trust Research Programme, P.O. Box 230, Kilifi, 80108 Kenya; 2 Centre for Biotechnology and Bioinformatics, University of Nairobi, Nairobi, Kenya; 3 Nuffield Department of Medicine, Centre for Clinical Vaccinology and Tropical Medicine, Churchill Hospital, University of Oxford, Oxford, UK; 4 Cambridge Institute for Medical Research, University of Cambridge, Hills Road, Cambridge, CB2 0XY UK

**Keywords:** Malaria, Vaccine, Rh5, CyRPA, Ripr, P113, Single nucleotide polymorphisms, Linkage disequilibrium

## Abstract

**Background:**

The invasion of the red blood cells by *Plasmodium falciparum* merozoites involves the interplay of several proteins that are also targets for vaccine development. The proteins *Pf*Rh5-*Pf*Ripr-*Pf*CyRPA-*Pf*p113 assemble into a complex at the apical end of the merozoite and are together essential for erythrocyte invasion. They have also been shown to induce neutralizing antibodies and appear to be less polymorphic than other invasion-associated proteins, making them high priority blood-stage vaccine candidates. Using available whole genome sequencing data (WGS) and new capillary sequencing data (CS), this study describes the genetic polymorphism in the Rh5 complex in *P. falciparum* isolates obtained from Kilifi, Kenya.

**Methods:**

162 samples collected in 2013 and 2014 were genotyped by capillary sequencing (CS) and re-analysed WGS from 68 culture-adapted *P. falciparum* samples obtained from a drug trial conducted from 2005 to 2007. The frequency of polymorphisms in the merozoite invasion proteins, PfRh5, PfRipr, PfCyRPA and PfP113 were examined and where possible polymorphisms co-occurring in the same isolates.

**Results:**

From a total 70 variants, including 2 indels, 19 SNPs [27.1%] were identified by both CS and WGS, while an additional 15 [21.4%] and 36 [51.4%] SNPs were identified only by either CS or WGS, respectively. All the SNPs identified by CS were non-synonymous, whereas WGS identified 8 synonymous and 47 non-synonymous SNPs. CS identified indels in repeat regions in the p113 gene in codons 275 and 859 that were not identified in the WGS data. The minor allele frequencies of the SNPs ranged between 0.7 and 34.9% for WGS and 1.1–29.6% for CS. Collectively, 12 high frequency SNPs (> 5%) were identified: four in Rh5 codon 147, 148, 203 and 429, two in p113 at codons 7 and 267 and six in Ripr codons 190, 259, 524, 985, 1003 and 1039.

**Conclusion:**

This study reveals that the majority of the polymorphisms are rare variants and confirms a low level of genetic polymorphisms in all proteins within the Rh5 complex.

**Supplementary Information:**

The online version contains supplementary material available at 10.1186/s12936-021-03815-x.

## Background

Despite some progress over the last decade, malaria continues to be a significant global health burden with a vaccine deemed essential to effectively control the disease in high malaria transmission zones [1]. The RTS,S vaccine has been rolled out in three African countries [2] but is < 50% protective [3], suggesting further iterations are required. There are other candidates in the pipeline that show promise for incorporation into second-generation vaccines. One leading candidate antigen is *Plasmodium falciparum* Reticulocyte Binding homologue 5 (Rh5, PF3D7_0424100), which is currently advancing through clinical trials [4].

Rh5 is the smallest in the Reticulocyte Binding Protein homolog (Rh) family that includes Rh1, Rh2a, Rh2b and Rh4 [5, 6]. Furthermore, it is the only member of the Rh family without a transmembrane domain. Rh5 has been shown to be refractory to gene knockout experiments, suggesting it plays an essential role in the invasion of erythrocytes [5, 6] via interactions with the erythrocyte receptor basigin (BSG) [7]. Both monoclonal and polyclonal anti-Rh5 antibodies inhibit erythrocyte invasion of multiple parasite strains by blocking the Rh5-BSG interaction in vitro [8–11]. Rh5 vaccination field trials in non-human primates, *Aotus* monkeys, demonstrated protection from heterologous *P. falciparum* challenge [12], while non-exposed vaccinated human volunteers from a phase 1a clinical trial, generated anti-Rh5 antibodies that blocked merozoite invasion in vitro [4]. Furthermore, while individuals from malaria endemic regions, who are naturally exposed to *P. falciparum* infections develop anti-PfRH5 antibodies at a relatively low prevalence, the presence of these antibodies have been associated with protection from symptomatic malaria in Papua New Guinea, and Mali [13–15]. Based on these findings, Rh5 has been considered as a next generation blood-stage malaria vaccine candidate even though it has low immunogenicity in natural infections.

Rh5 does not function in isolation during erythrocyte invasion, but acts as part of a multi-protein complex with Rh5 interacting protein (Ripr, PF3D7_0323400) [16], cysteine rich protein antigen (CyRPA, PF3D7_0423800) (17) and P113 (PF3D7_1420700) [18]. The Rh5-CyRPA-Ripr complex binds better to the erythrocyte cell surface than Rh5 alone [19], and interaction of Rh5 with its erythrocyte surface protein receptor, basigin, triggers a transient increase in Ca^2+^ concentration and alters the erythrocyte cytoskeleton [20]. Rh5 undergoes proteolytic cleavage, resulting in fragments of approximately 18 kDa and 45 kDa. Rh5 binds directly to P113 (via the smaller Rh5 fragment, [18] and CyRPA [17], while Ripr is associated with Rh5 through its interaction with CyRPA [21]. Therefore, CyRPA forms the contact sites for Rh5 and Ripr. It has been suggested that CyRPA dissociates from the complex and it is excluded from the membrane during binding to basigin. The Rh5-CyRPA-Ripr complex can bind to BSG without interaction with p113. However, P113 anchors Rh5 onto the merozoite membrane, while CyRPA and Ripr do not bind to erythrocytes on their own [16–18, 21].

Similar to Rh5, the genes encoding CyRPA and Ripr cannot be knocked out, suggesting that they are essential for parasite growth [16, 18], and conditional deletion of either Ripr and CyRPA results in non-invasive merozoites [19]. Antibodies to all three proteins (Rh5, CyRPA and Ripr) of the complex can inhibit erythrocyte invasion by multiple *P. falciparum* strains [16, 17, 22]. Furthermore, antibodies to CyRPA have been reported to block its interaction with the Rh5/Ripr complex and the formation of the multi-protein complex, leading to invasion inhibition [17]. In African and Papua New Guinean populations, P113 antibodies have been associated with protection against clinical malaria [13, 23]. All members of the Rh5 protein complex can, therefore, be considered potential blood-stage vaccine targets.

Polymorphisms are a particular barrier for the development of blood-stage vaccines, as proteins that are exposed to the immune system during invasion are often very diverse, presumably the result of pressure from the immune system [24]. This problem of diversity has impeded the development of blood-stage vaccines in the past, with AMA1 being a prime example. Like the Rh5 complex, AMA1 is essential for invasion, but it is highly polymorphic, resulting in immune responses that are allele-specific, a fact that may have limited the efficacy of previous Phase IIb trials [25]. However, Rh5, Ripr and CyRPA have been shown to be highly conserved [5, 22, 26], although polymorphisms in these genes including p113 have not been intensively investigated. In addition, exploring genetic diversity in all members of the complex in the same infections would identify whether polymorphisms are associated, which would need to be taken into consideration during vaccine design. To explore these questions, we examined all the four Rh5 complex genes by capillary and whole genome sequencing of a cross-sectional sample of parasites from Kilifi.

## Methods

### Sampling, DNA amplification and capillary sequencing

For capillary sequencing (CS), parasite DNA was extracted from 162 blood samples from children below 11 years admitted and attended to at the Kilifi County Hospital in 2013 and 2014. The children had variable parasitaemia ranging from 160 to 705,600 parasites/µl, with a median of 7440 parasites/µl. This study was reviewed and approved by the Centre Scientific Committee and the Scientific Ethical Review Unit (SERU) of the Kenya Medical Research Institute, on SERU protocol number 3149. *CyRPA* (PF3D7_0423800), *P113* (PF3D7_1420700), *Ripr* (PF3D7_0323400) and *Rh5* (PF3D7_0424100) genes were examined. Genomic DNA was previously extracted from packed frozen erythrocytes using the QIAcube (Qiagen), according to the manufacturer’s instructions (QIAGEN, UK). All four genes were amplified using High Fidelity Taq polymerase (Roche) (primers used are shown in Additional file 1: Table S1). PCR products were visualized on 1% agarose gels prior to sequencing to confirm their expected band size (Additional file 2: Table S2). Purified amplicons were directly sequenced using the PCR primers and additional sequencing primers (Additional file 1: Table 1), BigDye terminator chemistry v3.1 (Applied Biosystems, UK) and an ABI 3730xl capillary sequencer (Applied Biosystems, UK). The raw sequences for each targeted gene were assembled, edited, and aligned using SeqMan and MegAlign software (Lasergene 12; DNASTAR). All singleton SNP sites were confirmed by independent reamplification and resequencing of the relevant samples. Positions of the sequences that showed mixed or superimposed nucleotides (peak within a peak) were marked with IUPAC ambiguity codes and consider as a mixed infection and excluded from the SNP and haplotype frequencies.

### Sampling, DNA preparation and whole genome sequencing

For whole genome sequencing, parasite DNA was previously extracted from 68 blood samples obtained from children recruited into an artemisinin-based combination therapy (ACT) drug trial of dihydroartemisinin-piperaquine and artemether-lumefantrine conducted in Pingilikani dispensary, Kilifi from 2005 to 2007 [27]. Additionally, some samples were from patients admitted to the Kilifi County Hospital with severe malaria. All studies obtained clearance from the Kenya Medical Research Institute (KEMRI) Ethical Review Committee under protocol numbers SSC 945. Samples were cryopreserved in glycerolyte and later adapted to culture for about 2 months for chemosensitivity testing [28]. DNA was also extracted and contributed to MalariaGEN for whole genome sequencing (WGS) and genotyping on an Illumina Genome Analyzer to a read depth of approximately 98 × in genotyped sites, and reads of length 37–76 base pairs as described in Wendler et al*.* [29]. The genotype data generated from the sequence reads were obtained from the MalariaGEN *P. falciparum* Community Project [30]. The selected SNPs were from those identified in release 6.0.

### Read mapping and coverage analysis

A VCF file containing 68 samples obtained from Kilifi, Kenya, were used as the input file in the downstream analysis. Using VCFtools (v. 0.1.13) a targeted analysis of four genes: *Rh5, Ripr, CyRPA* and *P113* was filtered, by using a bed file containing the chromosome numbers and genomic positions, to generate one VCF file. Using PLINK [31], the VCF files were then examined to obtain a list of high quality SNPs, by excluding variants based on the following criteria: a) the SNPs with the ‘FAIL’ filter; b) non-coding SNPs; c) SNPs that have extremely low support (< 10 reads in one sample); and d) variants that did not pass the minor allele threshold of < 0.5% based on the number of reads obtained per variant.

### Global malariaGEN data retrieval and analysis

To further validate the SNPs, we identified through CS and WGS, data from the MalariaGEN *Plasmodium falciparum* community project version 4.0 was used. This data was generated through an analysis of 3488 *P. falciparum* samples collected at 43 different locations in West Africa (WAF), Central Africa (CAF), East Africa (EAF), South Asia (SAS), West South East Asia (WSEA), East South East Asia (ESEA), Oceanic (OCE) and South America (SAM). A total 930,000 exonic SNPs and their frequencies were obtained. The method used to generate the data are described in Amato et al*.* [32]. The dplyr v1.0.0 package [33] in R v4.0.2 [34] was used to filter our four genes of interest based on their unique Gene IDs: *CyRPA* (PF3D7_0423800), *P113* (PF3D7_1420700), *Ripr* (PF3D7_0323400) and *Rh5* (PF3D7_0424100). The pool of SNPs identified were filtered to obtain their frequencies.

### Population genetics statistical tests

The allele frequency distribution indices, Tajima’s D and Fu and Li’s D* and F*, were computed using DnaSP v5.10 software [35] for the capillary sequence data. Tajima’s D computed the differences between two estimators of theta, based on the number of segregating sites and the average number of nucleotide differences [36]. Fu and Li’s D* test statistic calculated the differences between the observed number of singletons (mutations appearing only once among the sequences), and the total number of mutations [37] Fu and Li’s F* test statistic considered the differences between the number of singletons and the average number of nucleotide differences between pairs of sequences [37]. For the p values DnaSP calculated the confidence limits of D (two-tailed test) and assumed that the statistic follows a beta distribution.

### Linkage disequilibrium analysis

For each of the four genes obtained from the whole genome data, the minor and major allele frequencies of all the SNPs were computed using PLINK. Only SNPs with a > 5% minor allele frequency were included in the analysis. The extent of linkage disequilibrium (LD) between pairs of SNPs in *Rh5, Ripr, CyRPA* and *P113* was determined within and between genes using R v3.6.0. The statistical significance of LD was tested, at the 5% level, using χ2 tests.

### Rh5-CyRPA-Ripr complex protein structures

The cryo-electron microscopy structure of Rh5-CyRPA-Ripr (PDB ID: 6MPV) was downloaded from the Protein Data Bank (http://www.rcsb.org/). Wong et al*.* [21] reported only the structures for Rh5 (residues 175–243 and 298–504) and CyRPA (residues 31–122, 126–242, 254–319 and 323–362) as the Ripr model could not be built de novo owing to resolution of the electron density map. However, based on the alpha helix structures described we use this to obtain a partial structure for Ripr. Using the generated dataset in this study, the Rh5 and CyRPA polymorphic sites were mapped onto their protein structures in Pymol (The PyMOL Molecular Graphics System, Version 2.2.0, Schrödinger, LLC), to determine the location of the polymorphisms in the three-dimensional conformation of the complex and whether the polymorphic sites were found in the binding regions of each protein.

## Results

### Population genetics summary statistics

All genes had a negative summary statistic, although only *P113* and *Ripr* reached significance with a negative value for either the Tajima’s D or Fu & Li D* & F* or both statistics (Tables 1 and 2). *P113* yielded values of -2.2, -3.2 and -3.4, respectively for CS data, with comparable results observed using the whole genome data of -2, -3.2 and -3.4. A similar observation was made with Ripr with the capillary data giving only significant values of − 2.5, and − 2.5 for the Fu & Li D* and F*, respectively, while the whole genome data yielded results of -2.8 and -2.9, respectively.

**Table 1:** Capillary sequence population genetics summary statistics

Gene	Size (bp)	n	S	Sn	π	Tajima's D	Fu & Li D*	Fu & Li F*
CyRPA 5'	586	45	1	1	0.00018	− 1.113	− 1.809	− 1.861
CyRPA 3'	225	87	4	2	0.00061	− 1.682	− 1.419	− 1.765
P113	2469	91	13	8	0.00019	− 2.22**	− 3.16*	-− 3.36^#^
Rh5	1105	50	7	2	0.0015	0.093	− 0.316	− 0.217
Ripr	1434	66	8	5	0.00056	− 1.349	− 2.479*	− 2.483*

n- number of sequences, S- number of segregating sites, Sn- number of singletons, *p < 0.05, **P < 0.01, #p < 0.02

**Table 2:** Whole genome sequence population genetics summary statistics

Gene	n	S	Sn	π	Tajima's D	Fu and Li's D*	Fu and Li's F*
CyRPA	68	5	2	0.00211	− 1.552	− 0.976	− 1.364
P113	68	12	8	0.00195	− 2.043*	− 3.240*	− 3.350**
Rh5	68	10	4	0.01171	− 0.376	− 1.272	− 1.092
Ripr	68	20	12	0.00956	− 1.648	− 2.847*	− 2.871*

n- number of sequences, S- number of segregating sites, Sn- number of singletons, *p < 0.05, **p < 0.02

### Genetic diversity in the Rh5 complex genes identified using capillary sequencing data

Capillary sequencing data was attempted from 162 samples taken from children admitted to Kilifi Hospital. Data on all four genes was not obtained by capillary sequencing for any single sample, but data was obtained for multiple pairs of genes from individual isolates: *p113* & *CyRPA*, *p113* & *Ripr* and *Rh5* & *CyRPA*. Capillary sequence data for both *p113* & *Rh5* and *CyRPA* & *Ripr* gene pairs were obtained in less than 20 samples, while 46 samples yielded sequence data for *P113* and *CyRPA* (Table 3). No synonymous SNPs were detected, while 32 non-synonymous (ns) SNPS were identified in total. *CyRPA*, *p113*, *Rh5* and *Ripr* sequences contained 4, 10, 6 and 12 SNPs, respectively (Table 4). Most SNPs were found in multiple isolates (Additional file 3), although 3 SNPs in *CyRPA* and *Ripr* and 5 in *p113* were singletons (found in only a single isolate). Indels were only found in p113, with variation in repeat regions at codon 275 with asparagine (N) (ranging from 3 to 9 N) and at codon 859 with glutamic acid (E) (ranging from 2 to 3E). The *CyRPA* analysis was conducted in two fragments from the N and C-terminal ends. The N-terminal end fragment, codons 1 to 170, contained only one non-synonymous SNP, at codon 165 and found in only a single infection, while the C-terminal end though shorter in comparison contained three polymorphic sites.

**Table 3:** The number of samples from which capillary sequence data was obtained for each gene combination

Genes	Samples (n = 162)
p113 + CyRPA	46
P113 + RIPR	25
Rh5 + CyRPA	24
p113 + Rh5	18
CyRPA + Ripr	14

**Table 4:** The frequency of variants in the Rh5 complex genes identified by CS and WGS

Gene	Position	S/NS/Indel	Frequency		Gene	Position	S/NS/Indel	Frequency	
			CS	WGS				CS	WGS
Rh5	Rh5_42	NS	2.3^#^	0	CyRPA	CyRPA_101	S	0	4.1
	Rh5_147*	NS	20.5	22.6		CyRPA_160*	NS	0	0.7^#^
	Rh5_148*	NS	20.5	22.6		CyRPA_165*	NS	2.2	0.7^#^
	Rh5_203*	NS	13.6	10.3		CyRPA_229	NS	0	1.4^#^
	Rh5_365*	NS	0	0.7^#^		CyRPA_235*	NS	0	0.7
	Rh5_371*	NS	0	4.1		CyRPA_236	NS	0	3.4^#^
	Rh5_389*	NS	0	0.7^#^		CyRPA_270*	NS	0	2.8
	Rh5_407*	NS	0	0.7^#^		CyRPA_271*	NS	2.3	0
	Rh5_410*	NS	4.6	3.4		CyRPA_292*	NS	1.2^#^	0
	Rh5_429*	NS	29.6	34.9		CyRPA_302*	NS	1.2^#^	2.1
	Rh5_439*^#^	NS	0	1.4		CyRPA_17*	NS	0	0.7^#^
P113	p113_5*	NS	0	1.4		CyRPA_19*	NS	0	0.7^#^
	p113_7*	S	0	5.1^#^	Ripr	RIPR_3	NS	0	1.4
	p113_234*	NS	1.1	1.4		RIPR_66*	NS	0	0.7^#^
	p113_267*	NS	4.4	7.5		RIPR_119	NS	0	0.7^#^
	p113_275_3N	Indel	2.2	0		RIPR_190*	NS	7.6	10.3
	p113_275_6N	Indel	72.5	0		RIPR_215*	NS	0	2.7
	p113_275_7N	Indel	1.1	0		RIPR_220	NS	1.5	0
	p113_275_8N	Indel	23.1	0		RIPR_226*	NS	3.0	0
	p113_275_9N	Indel	1.1	0		RIPR_228*	S	0	0.7^#^
	p113_310	NS	3.3	3.4		RIPR_255	NS	1.5	2.8
	p113_330*	NS	1.1^#^	0		RIPR_259*	NS	27.3	23.6
	p113_345*	S	0	1.4^#^		RIPR_327*	NS	4.6	3.4
	p113_381*	S	0	0.7^#^		RIPR_429	NS	0	0.7^#^
	p113_421*	NS	1.1^#^	0		RIPR_436*	NS	0	0.7^#^
	p113_453*	S	0	1.4^#^		RIPR_438*	NS	0	0.7^#^
	p113_478	NS	0	0.7^#^		RIPR_524*	NS	12.1	0.7^#^
	p113_485*	NS	0	1.4^#^		RIPR_564	NS	1.5^#^	0
	p113_620	NS	3.3	0		RIPR_587	NS	1.5	0
	p113_713*	NS	2.2	0		RIPR_744*	S	0	0.7^#^
	p113_716	NS	2.2^#^	0		RIPR_843	NS	0	1.4
	p113_750	S	0	0.7^#^		RIPR_858*	NS	0	0.7^#^
	p113_758*	NS	1.1	2.1		RIPR_883*	NS	0	0.7^#^
	p113_849	NS	1.1^#^	0		RIPR_985*	NS	12	8.9
	p113_859_2E	Indel	3.3	0		RIPR_995*	NS	0	1.4^#^
	p113_859_3E	Indel	96.7	0		RIPR_1003*	NS	15.4	10.3
	p113_912*	NS	0	1.4^#^		RIPR_1039*	NS	12	2.7
	p113_935*	NS	0	0.7^#^					

CyRPA: 12 SNPs, p113: 27 SNPs, Ripr: 25 SNPs and Rh5: 11 SNPs. SNPs that were identified by either CS or WGS and present in the global MalariaGEN dataset are indicated by an asterix* and singleton SNPs are indicated by a hash #

### Genetic diversity in the Rh5 complex genes identified using whole genome sequencing data

Whole genome sequencing (WGS) SNP data for all the four genes were obtained from 68 independent samples from a previous drug trial (Additional File 4). A total of 55 SNPs were identified within the *Rh5* gene complex: 10 in *CyRPA*, 14 in *P113,* 21 in Ripr and 10 in *Rh5* as shown in Table 2. There was a total of eight synonymous polymorphisms, 1 in *CyRPA*, 5 in *p113* and 2 in *Ripr* and forty-seven ns polymorphisms with *CyRPA*, *p113*, *Rh5* and *Ripr* containing 9, 9, 10 and 19, respectively (Table 4). Seven of the eight synonymous SNPs (unique to the WGS data) were singletons except *CyRPA* codon 101 whose frequency was > 5%. Given that our WGS data only contained SNP data, we did not explore the repeat sequences identified by CS in the p113 gene.

### Comparison of variants identified by CS and WGS with the global MalariaGEN data

In keeping with previous studies, the majority of variants in the Rh5 complex genes were rare, which meant that most were unique to each sequencing method, and very few SNPs were identified by both methods. In *Rh5*, *CyRPA*, *p113* and *Ripr* we observed 5, 1, 4, and 8 SNPs, respectively, that were identified by both methods. The MalariaGEN global variation dataset was screened to explore whether SNPs were missed, perhaps due to methodological differences. This analysis established that all common variants identified in our analysis (MAF > 5%) for *Rh5*, *P113* and *Ripr* were also found in the global MalariaGEN dataset, arguing against any systematic missing SNP identification issues. In addition, more than two thirds of the rare variant SNPs were identified in these samples had also been identified previously in the global MalariaGEN data, giving us confidence in the polymorphisms identified in this study using CS and WGS. Combining our data with global MalariaGEN data confirmed that the majority of variants in the genes encoding the *Rh5* complex are rare mutations (Table 4).

### Linkage disequilibrium analysis

The LD within and between genes was examined in SNPs with minor allele frequencies of > 5%. 6 SNPs and 4 SNPs were identified before and after Bonferroni correction, respectively. The 4 SNPs in LD after Bonferroni correction were within *Rh5* and *Ripr*. In *Rh5*, LD was observed in codons 147 and 148, p < 0.0001 and in Ripr, LD was observed between codons 985 and 1003 p-value of < 0.01. Due to the high number of rare variant SNPs in *CyRPA*, none were included in the LD analysis.

### Visualizing mutations on the protein complex structure

**Fig. 1:**
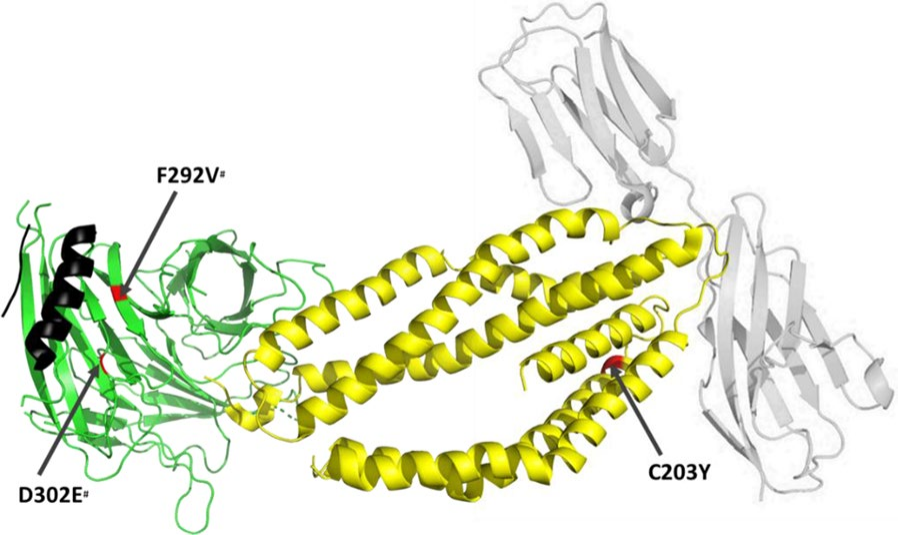
SNPs within protein–protein interacting regions of BSG, Rh5, CyRPA and Ripr. The interacting crystal structures of BSG (grey), Rh5 (yellow), CyRPA (green) and Ripr alpha helix (black) showing SNPs that fall within protein–protein interacting regions. In red, are the polymorphic residues identified in codon C203Y of Rh5 and codon D302E and F292V of CYPRA. Of the SNPs identified in the Rh5-CyRPA structure only the high frequency Rh5 codon 203 falls in the region that interacts with BSG. The singleton SNPs in codon F292V and D302E denoted by a hash#, lie within the region that interacts with Ripr. Apart from the short α-helical structure, the Ripr structure was not available on Protein Data Bank

Mutations within known protein interacting regions were mapped onto published structures for the Rh5 protein complex. The structure of the *CyRPA*-*Rh5* interaction has been published [21] and the Basigin structure was added to show its interaction with *Rh5*. The crystal structure of *Ripr* and *P113* has not been solved and hence their polymorphic residues in these proteins could not be mapped. *Rh5* interacts with BSG through an α-2, α-4 and a disulphide loop region [38]. This Rh5-basigin interacting region includes *Rh5* codon 203, a SNP that was identified at a frequency of > 5% in both the CS and WGS. We mapped back the identified rare variants to the protein structures (Fig. 1), and only two SNPs in *CyRPA* (codon 292 identified by CS and codon 302 identified by both CS and WGS, both at MAF < 5%) were located within the *CyRPA*-*Ripr* interacting region [21]. The *Ripr* α- helix, Fig. 1, corresponding to amino acid residues 196 – 211, interacts with blade 6 of the *CyRPA* β-propeller, amino acids 281 to 311 [21]. All the SNPs identified by CS and WGS in Ripr fall outside the Ripr α- helix, where the structure has not been solved.

## Discussion

The Rh5 complex is a relatively conserved set of proteins with few polymorphisms. They are not highly immunogenic, as previously shown [15, 23]. The negative population genetics summary statistics do not indicate balancing selection and show an excess of rare variants. This is consistent with an analysis of genomes from *P. falciparum* populations in Africa, which revealed that the majority of genes were associated with a negative Tajima’s D value. Therefore, suggesting there was a historical parasite population expansion in Africa [39–41]. The genes with a significant, negative population genetics summary statistics, indicate that these genes have a limited potential to retain mutations, in particular *p113* and *Ripr,* which may be due to the parasite’s need to preserve their function. These proteins are involved in a critical step during the invasion of erythrocytes and this polymorphism data reinforces the fact that they are likely to make good vaccine candidates to inhibit invasion and prevent disease [42].

Sequence data was obtained using two different methods and resulted in the identification of more SNPs using whole genome sequencing (WGS) analysis than Capillary Sequencing (CS), but there are pros and cons to both approaches. In CS, each read is accompanied by a long (on average 500 bp) chromatogram, which makes it easy to assemble and align to a reference genome in order to manually identify variants, but the process as a whole is low-throughput. In WGS, millions of short reads are produced with each read being accompanied by a quality score. It is thus not feasible to manually check the quality of each nucleotide and quality score cut-offs are set in the bioinformatic pipelines to confidently call a nucleotide. This presents a challenge in identifying indels within repeat regions—because the assembly and alignment of these regions to reference genomes is based on short reads, confidence is often low in these regions, making it difficult to unambiguously determine the numbers of repeat nucleotides [43]. However, the ability of WGS to generate large numbers of reads and identify SNPs in mixed infections allows more robust identification of SNPs, and it is therefore more reliable in the detection of low frequency variants as compared to CS. The Global MalariaGEN dataset was used to confirm the SNPs identified by the two methods. A large majority (> 65%) of the SNPs described in these samples have also been described in other locations within the Global MalariaGEN data, providing confidence both the high frequency and rare SNPs detected. Furthermore, most SNPs that were only identified by one method were rare variants, making it not surprising that there were missed by the other method, as the two methods were applied to different sample sets. If a rare variant is only present in few infections, the chances of such infections being present in the samples used for both methods is significantly reduced. It is also important to note that the samples utilized in WGS and CS, were obtained in different time points, which are 2005–2007 and 2013, respectively. In addition, the parasites used in obtaining the whole genome sequence data underwent culture-adaptation prior to sequencing, therefore the quality of DNA is expected to be higher in culture adapted parasites due to less contamination by host DNA. Cultured *P. falciparum* parasites have been known to differ significantly from source populations due to adaptation to environments that exclude the host immune responses [44]. There are therefore multiple reasons that could explain why different SNPs were identified in the two different approaches.

The majority of the polymorphisms in this complex or merozoite invasion antigens were rare, which is in contrast to previous findings from surface exposed and abundant merozoite antigens such as apical membrane antigen 1 (AMA1) [45], merozoite surface protein 1 (MSP1) [45], MSP3 [46] and erythrocyte binding antigen-175 (EBA175) [47], which are under balancing selection and exhibit allele-specific immunity in vaccine trials. In a recent study of samples from Nigeria, only 5 non-synonymous SNPs were identified in Rh5: K62R, T81Q, P197S, C203Y and H240R [48], of which only the C203Y mutation was identified in our study, while codon 197 was described in the global MalariaGEN dataset, codons 62, 81 and 240 are potentially rare variant sites. Of note, the high frequency sites of codons 147 and 148 in this study were not identified in the Nigerian study. However these aforementioned sites were described alongside codons S197Y, C203Y and I410M as common variants occurring at a frequency above 10% globally [9]. However, the I410M mutation was a rare variant (< 5%) in our population. It appears that apart from a few high frequency sites that have been consistently identified in previous studies and in our study, most mutations in Rh5 are rare variants. Rh5 antibodies primarily inhibit parasite invasion by disrupting the Rh5-basigin interaction [38].

This study identified only one Rh5 mutation C203Y at the Rh5-Basigin interface. It has been shown that the Rh5 protein variant with the 203Y mutant binds to recombinant basigin with the same affinity as the Rh5 C203 wild type [49]. It is therefore likely that other rare Rh5 mutations that cluster around the basigin interface will prevent binding of monoclonal antibodies. Based on monoclonal antibody data [50], these SNPs fall within the region of a large number of mouse and human antibodies that have shown neutralising activity within codons 26–352, suggesting that the rare variants identified in this study will potentially have an effect on antibody binding epitopes [9, 11, 17]. A similar scenario is observed with CyRPA, where only 1 SNP (R339S) was identified from a sample of 12 geographically distinct laboratory isolates and 6 field isolates [22] and again this SNP was not identified in the Kilifi samples. An analysis of 80 Ripr sequences from Uganda, identified 16 SNPs of which two codons (190 and 259) were > 5% in frequency. This study only found 9 of the 16 Ugandan SNPs and the SNPs unique to the Ugandan population were all singletons [26]. Moreover, Ntege et al. [26] also showed, like this study, a negative and significant Tajima’s D index. These studies further indicate that these genes tend to contain rare variants. The common variants identified across all the study sites should be considered in future studies to determine if they influence the functionality of the multiple protein complex.

The low immunogenicity of Rh5 complex members in field studies [12, 15, 22] would suggest limited immune pressure on these antigens and thus a limited need for the parasite to acquire mutations to escape host immune responses. This could explain the limited high frequency polymorphisms and the excess of rare variants observed. Slightly higher responses have been observed for p113 in individuals in Kilifi, when compared to Rh5 [23]. Beside the role of p113 in invasion by binding to the Rh5 N-terminal region [18], p113 is also thought to be involved in translocation through association with the *Plasmodium* translocon of exported proteins (PTEX), which is known to be a mechanism of immune evasion [51]. Further investigation is required to understand the effect of P113 polymorphisms on translocation. While there is limited literature on natural immune responses to Ripr, we anticipate similar findings as seen with CyRPA and Rh5, given that Ripr is part of the same Rh5 protein complex. The Rh5 protein complex is hidden within the merozoite apical end during tight junction formation. It is, therefore, likely that these proteins are rarely exposed to the immune system and thus their immunogenicity in individuals living in malaria endemic regions is low. Their role in tight junction formation indicates an important function in merozoite invasion, which has been determined by an inability to genetically disrupt all of the 4 genes and by the protective immune responses generated by antigens like Rh5 and p113 [50].

Most of the observed SNPs were not in statistically significant LD with the exception of codons 147 and 148 for Rh5 and 985 and 1003 in CyRPA, which are 3 bp and 54 bp apart respectively. The limited LD is likely due to a combination of the fact that most of the SNPs are rare variants and therefore occur at a low frequency, and the limited sample size in this study. Rh5 codons, 147 and 148 are included in the protein structure [38] on the upstream of the alpha helix, while the structure of Ripr has not been fully resolved. Since they are high frequency SNPs, they may be involved in processes other than protein-proteins interactions, but these are yet to be determined. Only one high frequency SNP at Rh5 codon 203, identified by both CS and WGS, has been shown to be localized in the Rh5-basigin interface [38].

The development of new tools and adaptation of existing tools for use in malaria elimination and eradication remains a priority, and deeper understanding of polymorphism(s) in vaccine candidate genes is particularly important. This study highlights pros and cons to both CS and WGS approaches to identifying vaccine-relevant polymorphisms. The ideal molecular tool should be able to provide quality and high-throughput sequence reads capable of detecting low frequency variants including indels. One such approach would be amplicon deep sequencing, where longer fragment amplicons can be generated and sequenced using an NGS platform, focussing analysis on the regions of interest rather than the whole genome, but producing deeper and higher quality data than CS. Low frequency mutations should be assessed by functional assays to ascertain their biological and immunological relevance. One of the main obstacles in the development of effective vaccines for malaria is the occurrence of polymorphisms on candidate vaccine targets that result in strain-specific immunity. Among the members of the Rh5 complex, Rh5 is the most advanced in vaccine development. The identification of a limited number of high frequency polymorphisms on Rh5 shows promising prospects of Rh5 based vaccines in this region, but it is still possible that low frequency variants may lead to immune evasion—this needs to be systematically investigated.

## Conclusion

One gene does not appear to conceal the other genes in the complex, by being more polymorphic and acting as a decoy to direct the immune pressure away from the rest of the genes in the complex. Thus, the limited polymorphisms are potentially a result of their hidden location in the apical end of the merozoite and their limited exposure to host immune responses. Due to the minimal acquisition of mutations, Rh5, CyRPA, Ripr and P113 proteins are potentially a good next-generation multi-antigen vaccine formulation.

## Supplementary Information

**Additional file 1: Table S1.** List of primers for PCR and capillary sequencing.

**Additional file 2: Table S2.** The expected PCR region amplified and product size for each gene.

**Additional file 3: Table S3.** List of SNPs identified by capillary electrophoresis method.

**Additional file 4: Table S4.** List of SNPs identified by whole genome sequencing method.

## Data Availability

The DNA sequence data for Rh5, Ripr, CyRPA and p113 genes were deposited in GenBank and are available under the accession codes for P113: MW597459—MW597549, Rh5: MW597550—MW597609, CyRPA: MW597610—MW597716, Ripr: MW597717—MW597740.

## References

[1] WHO. World Malaria Report [Internet]. Geneva, World Health Organization. 2019 [cited 2020 Jan 10]. p. 238. https://www.who.int/publications-detail/world-malaria-report-2019

[2] Adepoju P (2019). RTS, S malaria vaccine pilots in three African countries. Lancet.

[3] RTS,S Clinical Trials Partnership. Efficacy and safety of RTS,S/AS01 malaria vaccine with or without a booster dose in infants and children in Africa: final results of a phase 3, individually randomised, controlled trial. Lancet. 2015. 386:31–45. 10.1016/S0140-6736(15)60721-8 PMC5626001 25913272

[4] Payne RO, Silk SE, Elias SC, Miura K, Diouf A, Galaway F (2017). Human vaccination against RH5 induces neutralizing antimalarial antibodies that inhibit RH5 invasion complex interactions. JCI Insight.

[5] Hayton K, Gaur D, Liu A, Takahashi J, Henschen B, Singh S (2008). Erythrocyte binding protein PfRH5 polymorphisms determine species-specific pathways of *Plasmodium falciparum* invasion. Cell Host Microbe.

[6] Baum J, Chen L, Healer J, Lopaticki S, Boyle M, Triglia T (2009). Reticulocyte-binding protein homologue 5 - An essential adhesin involved in invasion of human erythrocytes by *Plasmodium falciparum*. Int J Parasitol.

[7] Crosnier C, Bustamante LY, Bartholdson SJ, Bei AK, Theron M, Uchikawa M (2011). Basigin is a receptor essential for erythrocyte invasion by *Plasmodium falciparum*. Nature.

[8] Douglas AD, Williams AR, Illingworth JJ, Kamuyu G, Biswas S, Goodman AL (2011). The blood-stage malaria antigen PfRH5 is susceptible to vaccine-inducible cross-strain neutralizing antibody. Nat Commun.

[9] Bustamante LY, Bartholdson SJ, Crosnier C, Campos MG, Wanaguru M, Nguon C (2013). A full-length recombinant *Plasmodium falciparum* PfRH5 protein induces inhibitory antibodies that are effective across common PfRH5 genetic variants. Vaccine.

[10] Douglas AD, Williams AR, Knuepfer E, Illingworth JJ, Furze JM, Crosnier C (2014). Neutralization of *Plasmodium falciparum* merozoites by antibodies against PfRH5. J Immunol.

[11] Alanine DGW, Quinkert D, Kumarasingha R, Mehmood S, Donnellan FR, Minkah NK (2019). Human antibodies that slow erythrocyte invasion potentiate malaria-neutralizing antibodies. Cell.

[12] Douglas AD, Baldeviano GC, Lucas CM, Lugo-Roman LA, Crosnier C, Bartholdson SJ (2015). A PfRH5-based vaccine is efficacious against heterologous strain blood-stage *Plasmodium falciparum* infection in *Aotus* monkeys. Cell Host Microbe.

[13] Richards JS, Arumugam TU, Reiling L, Healer J, Hodder AN, Fowkes FJI (2013). Identification and prioritization of merozoite antigens as targets of protective human immunity to *Plasmodium falciparum* malaria for vaccine and biomarker development. J Immunol.

[14] Patel SD, Ahouidi AD, Bei AK, Dieye TN, Mboup S, Harrison SC (2013). *Plasmodium falciparu*m merozoite surface antigen, PfRH5, elicits detectable levels of invasion-inhibiting antibodies in humans. J Infect Dis.

[15] Tran TM, Ongoiba A, Coursen J, Crosnier C, Diouf A, Huang CY (2014). Naturally acquired antibodies specific for *Plasmodium falciparum* reticulocyte-binding protein homologue 5 inhibit parasite growth and predict protection from malaria. J Infect Dis.

[16] Chen L, Lopaticki S, Riglar DT, Dekiwadia C, Uboldi AD, Tham W-H (2011). An EGF-like protein forms a complex with PfRh5 and is required for invasion of human erythrocytes by *Plasmodium falciparum*. PLoS Pathog.

[17] Reddy KS, Amlabu E, Pandey AK, Mitra P, Chauhan VS, Gaur D (2015). Multiprotein complex between the GPI-anchored CyRPA with PfRH5 and PfRipr is crucial for *Plasmodium falciparum* erythrocyte invasion. Proc Natl Acad Sci USA.

[18] Galaway F, Drought LG, Fala M, Cross N, Kemp AC, Rayner JC (2017). P113 is a merozoite surface protein that binds the N terminus of *Plasmodium falciparum* RH5. Nat Commun.

[19] Volz JC, Yap A, Sisquella X, Thompson JK, Lim NTY, Whitehead LW (2016). Essential role of the PfRh5/PfRipr/CyRPA complex during *Plasmodium falciparum* invasion of erythrocytes. Cell Host Microbe.

[20] Aniweh Y, Gao X, Hao P, Meng W, Lai SK, Gunalan K (2017). P falciparum RH5-Basigin interaction induces changes in the cytoskeleton of the host RBC. Cell Microbiol.

[21] Wong W, Huang R, Menant S, Hong C, Sandow JJ, Birkinshaw RW (2019). Structure of *Plasmodium falciparum* Rh5–CyRPA–Ripr invasion complex. Nature.

[22] Dreyer AM, Matile H, Papastogiannidis P, Kamber J, Favuzza P, Voss TS (2012). Passive immunoprotection of *Plasmodium falciparum* -Infected mice designates the CyRPA as candidate malaria vaccine antigen. J Immunol.

[23] Osier FH, Mackinnon MJ, Crosnier C, Fegan G, Kamuyu G, Wanaguru M (2014). New antigens for a multicomponent blood-stage malaria vaccine. Sci Transl Med.

[24] Genton B, Betuela I, Felger I, Al-Yaman F, Anders RF, Saul A (2002). A recombinant blood-stage malaria vaccine reduces *Plasmodium falciparum* density and exerts selective pressure on parasite populations in a Phase 1–2b trial in Papua New Guinea. J Infect Dis.

[25] Sagara I, Dicko A, Ellis RD, Fay MP, Diawara SI, Assadou MH (2009). A randomized controlled phase 2 trial of the blood stage AMA1-C1/Alhydrogel malaria vaccine in children in Mali. Vaccine.

[26] Ntege EH, Arisue N, Ito D, Hasegawa T, Palacpac NMQ, Egwang TG (2016). Identification of *Plasmodium falciparum* reticulocyte binding protein homologue 5-interacting protein, PfRipr, as a highly conserved blood-stage malaria vaccine candidate. Vaccine.

[27] Borrmann S, Sasi P, Mwai L, Bashraheil M, Abdallah A, Muriithi S (2011). Declining responsiveness of *Plasmodium falciparum* infections to artemisinin-based combination treatments on the Kenyan coast. PLoS ONE.

[28] Sasi P, Abdulrahaman A, Mwai L, Muriithi S, Straimer J, Schieck E (2009). In vivo and in vitro efficacy of amodiaquine against *Plasmodium falciparum* in an area of continued use of 4-aminoquinolines in East Africa. J Infect Dis.

[29] Wendler JP, Okombo J, Amato R, Miotto O, Kiara SM, Mwai L (2014). A genome wide association study of *Plasmodium falciparum* susceptibility to 22 antimalarial drugs in Kenya. PLoS ONE.

[30] Manske M, Miotto O, Campino S, Auburn S, Almagro-Garcia J, Maslen G (2012). Analysis of *Plasmodium falciparum* diversity in natural infections by deep sequencing. Nature.

[31] Chang CC, Chow CC, Tellier LC, Vattikuti S, Purcell SM, Lee JJ (2015). Second-generation PLINK: rising to the challenge of larger and richer datasets. Gigascience.

[32] Amato R, Miotto O, Woodrow CJ, Almagro-Garcia J, Sinha I, Campino S (2016). Genomic epidemiology of artemisinin resistant malaria. Elife..

[33] Wickham H, François R, Henry L, Müller K. dplyr: a grammar of data manipulation Version 1.0.2. [Internet]. 2020 https://dplyr.tidyverse.org/

[34] Team RC. The R Project for Statistical Computing. [Internet]. 2013. [cited 2020 Aug 5];1–12. https://www.r-project.org/

[35] Rozas J, Sánchez-delbarrio JC, Messeguer X, Rozas R (2003). DnaSP, DNA polymorphism analyses by the coalescent and other methods. Bioinformatics.

[36] Tajima F (1989). Statistical method for testing the neutral mutation hypothesis by DNA polymorphism. Genetics.

[37] Li W (1993). Statistical tests of neutrality of mutations. Genetics.

[38] Wright KE, Hjerrild KA, Bartlett J, Douglas AD, Jin J, Brown RE (2014). Structure of malaria invasion protein RH5 with erythrocyte basigin and blocking antibodies. Nature.

[39] Amambua-Ngwa A, Tetteh KKA, Manske M, Gomez-Escobar N, Stewart LB, Deerhake ME (2012). Population genomic scan for candidate signatures of balancing selection to guide antigen characterization in malaria parasites. PLoS Genet.

[40] Mobegi VA, Duffy CW, Amambua-Ngwa A, Loua KM, Laman E, Nwakanma DC (2014). Genome-wide analysis of selection on the malaria parasite *Plasmodium falciparum* in West African populations of differing infection endemicity. Mol Biol Evol.

[41] Ocholla H, Preston MD, Mipando M, Jensen ATR, Campino S, Macinnis B (2014). Whole-genome scans provide evidence of adaptive evolution in Malawian P*lasmodium falciparum* isolates. J Infect Dis.

[42] Draper SJ, Sack BK, King CR, Nielsen CM, Rayner JC, Higgins MK (2018). Malaria vaccines: recent advances and new horizons. Cell Host Microbe.

[43] Tørresen OK, Star B, Mier P, Andrade-Navarro MA, Bateman A, Jarnot P (2019). Tandem repeats lead to sequence assembly errors and impose multi-level challenges for genome and protein databases. Nucleic Acids Res.

[44] Claessens A, Affara M, Assefa SA, Kwiatkowski DP, Conway DJ (2017). Culture adaptation of malaria parasites selects for convergent loss-of-function mutants. Sci Rep.

[45] Polley SD, Conway DJ (2001). Strong diversifying selection on domains of the *Plasmodium falciparum* apical membrane antigen 1 gene. Genetics.

[46] Polley SD, Tetteh KKA, Lloyd JM, Akpogheneta OJ, Greenwood BM, Bojang KA (2007). *Plasmodium falciparum* merozoite surface protein 3 is a target of allele-specific immunity and alleles are maintained by natural selection. J Infect Dis.

[47] Verra F, Chokejindachai W, Weedall GD, Polley SD, Mwangi TW, Marsh K (2006). Contrasting signatures of selection on the *Plasmodium falciparum* erythrocyte binding antigen gene family. Mol Biochem Parasitol.

[48] Ajibaye O, Osuntoki AA, Balogun EO, Olukosi YA, Iwalokun BA, Oyebola KM (2020). Genetic polymorphisms in malaria vaccine candidate *Plasmodium falciparum* reticulocyte-binding protein homologue-5 among populations in Lagos. Nigeria Malar J.

[49] Hjerrild KA, Jin J, Wright KE, Brown RE, Marshall JM, Labbé GM (2016). Production of full-length soluble *Plasmodium falciparum* RH5 protein vaccine using a *Drosophila melanogaster* Schneider 2 stable cell line system. Sci Rep.

[50] Ragotte RJ, Higgins MK, Draper SJ (2020). The RH5-CyRPA-Ripr complex as a malaria vaccine target. Trends Parasitol.

[51] Elsworth B, Sanders PR, Nebl T, Batinovic S, Kalanon M, Nie CQ (2016). Proteomic analysis reveals novel proteins associated with the *Plasmodium* protein exporter PTEX and a loss of complex stability upon truncation of the core PTEX component, PTEX150. Cell Microbiol.

