# Supplementary material for: The Plasmodium falciparum Rh5 invasion protein complex reveals an excess of rare variant mutations
Source: Malar J. 2021 Jun 23;20:278. doi: 10.1186/s12936-021-03815-x (PMC8220363; doi:10.1186/s12936-021-03815-x)
Supplement: Supplementary file 1 — Additional file 1: Table S1. List of primers for PCR and capillary sequencing. [file 12936_2021_3815_MOESM1_ESM.docx]

Additional Table 1: List of Primers for PCR and Capillary Sequencing

| **Primer ID** | **Sequence (5' - 3' Orientation)** |
| --- | --- |
| **Ripr_F3** | ATGTGCTTATGGAAATACA |
| **Ripr_F4** | ACATGCTATGGGAACAG |
| **Ripr_F6*** | ACAAATACGTTTATACTATATTAAATCGT |
| **Ripr_R1*** | CTAATTCTGATTACTATAATAAAATAC |
| **Ripr_R2** | TAGCACATATACACACACCATTCT |
| **Ripr_R3** | TGATGGTATATTAGAATGA |
| **CyprA_F1*** | TGAAAATAACATTATGATTATCCCT |
| **CyprA_F3*** | TGAGTGTACCCATGAAAAGGA |
| **CyprA_R1*** | TCCTTGCAGTAACCCCTTTTGTCTAC |
| **CyprA_R2*** | TGTCATCCTTCTTATTGTCATCCT |
| **Pf113_F1*** | ATGTGTTCACCTTTTAGATGTTCAC |
| **Pf113_R1*** | ACTAAGAGTGCATTACTCATAGCAAAGA |
| **Pf113_F2** | AGCTGATAATATATCCTTATTATTG |
| **Pf113_F3** | CACATGAAAGAATACTTAAAAAATTAC |
| **Pf113_R2** | TGTGCTTTTTCATATAGGGCAT |
| **Pf113_R3** | TGCATCACGTTTCATTTCATTATATC |
| **Rh5_F*** | CGAAGAATCAAGAAAATAATCTG |
| **RH5_OR*** | ATGTTTTGTCATTTCATTG |
| **Rh5_R2** | TCTTCGGTTTCATCATCTGT |
| **Rh5_R** | GAATATTCATTTGACATGTC |

All primers were used for sequencing while those marked with * were used for both PCR and Sequencing.
