# Supplementary material for: The Plasmodium falciparum Rh5 invasion protein complex reveals an excess of rare variant mutations
Source: Malar J. 2021 Jun 23;20:278. doi: 10.1186/s12936-021-03815-x (PMC8220363; doi:10.1186/s12936-021-03815-x)
Supplement: Supplementary file 2 — Additional file 2: Table S2. The expected PCR region amplified and product size for each gene. [file 12936_2021_3815_MOESM2_ESM.docx]

Additional Table 2: The expected region amplified and PCR product size for each gene

| **Gene** | **Gene size (bp)** | **Region amplified** | **PCR Anneal temperature** | **PCR primers** | **PCR product size (bp)** | **Sequencing primers** |
| --- | --- | --- | --- | --- | --- | --- |
| **Ripr** | 3261 | full length | 54^a^ | F6/R1 | 3064 | F3, F4, F6, R1, R2, R3 |
| **CyRPA** | 1188 | exon 1 | 44* | F1/R2 | 586 | F1, R2 |
|  |  | exon 2 | 51* | F3/R1 | 313 | F3, R1 |
| **P113** | 3070 | exon 3 | 63^ | F1/R1 | 2743 | F1, F2, F3, R1, R2, R3 |
| **Rh5** | 1788 | exon 2 | 54* | F/OR | 1200 | F, OR, R, R2 |

^a^10 cycles (94°C for 2min, 94°C for 15s, anneal temp for 30s, 72°C for 2min) and 25cycles (94°C for 15s, anneal temp for 30s, 72°C for 2min + 5s/cycle) final elongation 72°C for 7min

*10 cycles (94°C for 2min, 94°C for 15s, anneal temp for 30s, 72°C for 45s) and 25cycles (94°C for 15s, anneal temp for 30s, 72°C for 45s + 5s/cycle) final elongation 72°C for 7min

^10 cycles (94°C for 2min, 94°C for 30s, anneal temp for 30s, 68°C for 2min) and 25cycles (94°C for 30s, anneal temp for 30s, 68°C for 2min + 5s/cycle) final elongation 72°C for 5min
