# Supplementary material for: The Plasmodium falciparum Rh5 invasion protein complex reveals an excess of rare variant mutations
Source: Malar J. 2021 Jun 23;20:278. doi: 10.1186/s12936-021-03815-x (PMC8220363; doi:10.1186/s12936-021-03815-x)
Supplement: Supplementary file 3 — Additional file 3: Table S3. List of SNPs identified by capillary electrophoresis method. [file 12936_2021_3815_MOESM3_ESM.docx]

Additional Table 4: List of SNPs identified by Capillary Electrophoresis method

| **Gene_ID, Gene** | **Position** | **REF** | **ALT** | **MAF** | **Nucleotide** | **Codon** | **Synonymous (S)/ Non-synonymous (NS)/Indel** |
| --- | --- | --- | --- | --- | --- | --- | --- |
| **Pf3D7_0424100, Rh5** | 1083819 | L | V | 2.27 | 125 | 42 | NS |
|  | 1083505 | A | G | 20.45 | 439 | 147 | NS |
|  | 1083502 | G | C | 20.45 | 442 | 148 | NS |
|  | 1083336 | C | T | 13.6 | 608 | 203 | NS |
|  | 1082714 | T | C | 4.6 | 1230 | 410 | NS |
|  | 1082657 | T | G | 29.6 | 1287 | 429 | NS |
| **Pf3D7_0423800, CyRPA** | 1076839 | G | A | 2.2 | 493 | 165 | NS |
|  | 1077258 | R | S | 2.3 | 812 | 271 | NS |
|  | 1077319 | V | F | 1.2 | 875 | 292 | NS |
|  | 1077351 | G | C | 1.2 | 906 | 302 | NS |
| **PF3D7_0323400, Rh5 interacting protein (RIPR)** | 983398 | A | G | 7.6 | 569 | 190 | NS |
|  | 983308 | D | N | 1.5 | 659 | 220 | NS |
|  | 983291 | T | H | 3.03 | 677 | 226 | NS |
|  | 983202 | C | T | 1.5 | 765 | 255 | NS |
|  | 983192 | A | G | 27.3 | 775 | 259 | NS |
|  | 982988 | T | C | 4.6 | 979 | 327 | NS |
|  | 982396 | T | A | 12.1 | 1571 | 524 | NS |
|  | 982276 | E | Q | 1.5 | 1691 | 564 | NS |
|  | 982207 | I | T | 1.5 | 1760 | 587 | NS |
|  | 981014 | A | T | 12 | 2953 | 985 | NS |
|  | 980960 | C | A | 15.4 | 3007 | 1003 | NS |
|  | 980850 | T | C | 12 | 3117 | 1039 | NS |
| **Pf3D7_1420700, surface protein P113** | 860870 | G | A | 1.1 | 700 | 234 | NS |
|  | 860970 | A | C | 4.4 | 800 | 267 | NS |
|  | 861099 | G | T | 3.3 | 929 | 310 | NS |
|  | 861158 | E | K | 1.1 | 989 | 330 | NS |
|  | 861431 | A | V | 1.1 | 1262 | 421 | NS |
|  | 862029 | Q | H | 3.3 | 1859 | 620 | NS |
|  | 862309 | Q | E | 2.2 | 2138 | 713 | NS |
|  | 862317 | N | H | 2.2 | 2147 | 716 | NS |
|  | 862443 | A | G | 1.1 | 2273 | 758 | NS |
|  | 862716 | Q | K | 1.1 | 2546 | 849 | NS |
|  | 860994 | 6N | 3N | 2.2 | 824 | 275 | indel |
|  | 860994 | 6N | 7N | 1.1 | 824 | 275 | indel |
|  | 860994 | 6N | 8N | 23.1 | 824 | 275 | indel |
|  | 860994 | 6N | 9N | 1.1 | 824 | 275 | indel |
|  | 862746 | 3E | 2E | 3.3 | 2576 | 859 | indel |
|  |  |  |  |  |  |  |  |
|  |  |  |  |  |  |  |  |

REF: the 3D7 reference allele, ALT: the alternative allele, MAF: minor allele frequency
