# Supplementary material for: The Plasmodium falciparum Rh5 invasion protein complex reveals an excess of rare variant mutations
Source: Malar J. 2021 Jun 23;20:278. doi: 10.1186/s12936-021-03815-x (PMC8220363; doi:10.1186/s12936-021-03815-x)
Supplement: Supplementary file 4 — Additional file 4: Table S4. List of SNPs identified by whole genome sequencing method. [file 12936_2021_3815_MOESM4_ESM.docx]

Additional Table 3: List of SNPs identified by Whole Genome Sequencing method

| **Gene_ID, Gene** | **Position** | **REF** | **ALT** | **MAF** | **Nucleotide** | **Codon** | **Synonymous (S)/ Non-synonymous (NS)** |
| --- | --- | --- | --- | --- | --- | --- | --- |
| **PF3D7_0323400, Rh5 interacting protein (RIPR)** | 980850 | T | C | 2.74 | 3117 | I1039M | NS |
|  | 980960 | C | A | 10.27 | 3007 | A1003S | NS |
|  | 980982 | G | T | 1.37 | 2985 | D995E | NS |
|  | 981014 | A | T | 8.904 | 2953 | Y985N | NS |
|  | 981320 | C | A | 0.6849 | 2647 | V883L | NS |
|  | 981394 | G | A | 0.6849 | 2573 | S858F | NS |
|  | 981439 | T | C | 1.37 | 2528 | E843G | NS |
|  | 981735 | A | G | 0.6849 | 2232 | R744 | S |
|  | 982396 | T | A | 0.6849 | 1571 | H524L | NS |
|  | 982654 | T | C | 0.6849 | 1313 | D438G | NS |
|  | 982661 | T | C | 0.6849 | 1306 | M436V | NS |
|  | 982682 | C | G | 0.6849 | 1285 | V429L | NS |
|  | 982988 | T | C | 3.425 | 979 | T327A | NS |
|  | 983192 | A | G, T | 23.61 | 775 | Y259H/N | NS |
|  | 983202 | C | T, G | 2.778 | 765 | M255I | NS |
|  | 983283 | A | C | 0.6849 | 684 | N228 | S |
|  | 983322 | G | T | 2.74 | 645 | N215K | NS |
|  | 983398 | A | G | 10.27 | 569 | V190A | NS |
|  | 983611 | C | G | 0.6849 | 356 | G119A | NS |
|  | 983771 | T | A | 0.6849 | 196 | N66Y | NS |
|  | 983959 | C | G | 1.389 | 8 | R3T | NS |
| **Pf3D7_0423800, CyRPA** | 1076397 | A | C | 0.6944 | 51 | L17F | NS |
|  | 1076401 | G | T | 0.6944 | 55 | V19F | NS |
|  | 1076649 | C | T | 4.11 | 303 | G101 | S |
|  | 1076824 | C | A | 0.6944 | 478 | L160I | NS |
|  | 1076839 | G | A | 0.6944 | 493 | V165I | NS |
|  | 1077132 | A | C | 1.37 | 687 | R229S | NS |
|  | 1077150 | C | T | 0.6849 | 705 | N235K | NS |
|  | 1077151 | G | A | 3.425 | 706 | D236N | NS |
|  | 1077254 | A | C | 2.778 | 809 | N270T | NS |
|  | 1077351 | G | C | 2.055 | 906 | E302D | NS |
| **Pf3D7_0424100, Rh5** | 1082627 | T | C | 1.37 | 1317 | Q439K | NS |
|  | 1082657 | T | G | 34.93 | 1287 | K429N | NS |
|  | 1082714 | T | C | 3.425 | 1230 | I410M | NS |
|  | 1082725 | T | C | 0.6849 | 1219 | I407V | NS |
|  | 1082779 | G | T | 0.6849 | 1165 | Q389K | NS |
|  | 1082833 | C | T | 4.11 | 1111 | V371I | NS |
|  | 1082851 | G | T | 0.6849 | 1093 | H365N | NS |
|  | 1083336 | C | T | 10.27 | 608 | C203Y | NS |
|  | 1083502 | G | C | 22.6 | 442 | H148D | NS |
|  | 1083505 | A | G | 22.6 | 439 | Y147H | NS |
| **Pf3D7_1420700, surface protein P113** | 860025 | T | A | 1.449 | 15 | F5L | NS |
|  | 860031 | T | A | 5.072 | 21 | 7I | S |
|  | 860136 | A | G | 5.072 |  | INTRON |  |
|  | 860870 | G | A | 1.37 | 700 | E234K | NS |
|  | 860970 | A | C | 7.534 | 800 | D267A | NS |
|  | 861099 | G | T | 3.425 | 929 | G310V | NS |
|  | 861205 | A | G | 1.37 | 1035 | 345K | S |
|  | 861313 | A | G | 0.6849 | 1143 | 381R | S |
|  | 861529 | T | A | 1.37 | 1359 | 453T | S |
|  | 861604 | G | A | 0.6849 | 1434 | M478I | NS |
|  | 861624 | T | A | 1.37 | 1454 | F485Y | NS |
|  | 862420 | A | G | 0.6849 | 2250 | 750E | S |
|  | 862443 | A | G | 2.055 | 2273 | D758G | NS |
|  | 862905 | A | G | 1.37 | 2735 | E912G | NS |
|  | 862974 | C | T | 0.6849 | 2804 | T935M | NS |

REF: the 3D7 reference allele, ALT: the alternative allele, MAF: minor allele frequency
